# Ultra-thin Rigid diagnostic and therapeutic arthroscopy during arthrocentesis: Development and preliminary clinical findings

**DOI:** 10.1186/s40902-015-0018-0

**Published:** 2015-07-15

**Authors:** Seong-Yong Moon, Hoon Chung

**Affiliations:** 1 Department of Oral and Maxillofacial Surgery, School of Dentistry, Chosun University, Gwang-Ju, South Korea; 2 Hoon Chung Dental Clinic, #2305, Jangkyo BLDG.,1 Jangkyo-Dong, Seoul, Jung-Gu Zip Code: 110-760 Republic of Korea

**Keywords:** Arthroscopy, Arthocentesis, TMD, Closed lock, Anterior disc displacement without reduction, Habitual dislocation

## Abstract

Arthroscopy is useful to detect early changes in the temporomandibular joint (TMJ).

Despite great advances in arthroscopy, many arthroscopic surgeries have now been replaced by arthrocentesis. We propose a simple diagnostic and therapeutic method having operative rigid ultra-thin arthroscopy with 16 gauge needle size combined with arthrocentesis.

## Background

Arthroscopy of the temporomandibular joint (TMJ) was first reported by Ohnishi [1]. Arthroscopy is useful to detect early changes of intra-articular space in the temporomandibular joint (TMJ) that can not be detected with magnetic resonance imaging (MRI) and computed tomography (CT) [2].

Arthrocentesis is regarded as being less invasive than arthroscopic lysis and lavage. In addition, the effectiveness of arthrocentesis has been shown to be clinically acceptable [3] and not different from that of arthroscopic surgery. However, conventional TMJ arthrocentesis does not provide any information in joint pathosis [4]. Operative arthroscopy provides benefits over arthrocentesis because of the ability to visualize the joint for diagnostic purposes and perform surgical maneuvers and it offers a good success rate [5]. Recently fiber ultra-thin arthroscopy is developed which is useful and valuable in examination of the pathologic TMJ, however the resolution of the view is unclear [6].

In this report, we propose a simple diagnostic and therapeutic method of operative ultra-thin rigid arthroscopy combination with arthrocentesis through the Chung's needle (16 gauge needle size).

### Chung’s needle and Arthroscopy

1. Chung's needle (Fig. 1)

**Fig. 1:**
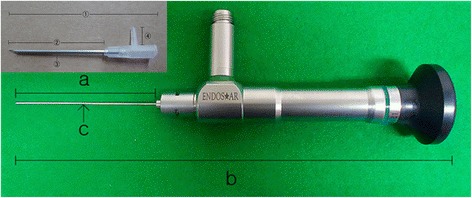
Chung’s needle has 66.20 mm of total length (1), 43.50 mm of available length (2), 1.65 mm of exteranl diameter (3), and 8.30 mm of irrigation port (4). The ultra-thin rigid arthroscopy (Endostar-Nanoscopy System (Hanseo Medics Co., Ltd., Seoul, Korea)) consists of 167 mm of total length (**b**), 57.5 mm of available length (**a**), a 0.8 mm diameter fiber optic nanoscope (**c**)
  Chung’s needle has 66.20 mm of total length, 43.50 mm of available length, 1.65 mm of external diameter, and 8.30 mm of irrigation port. Rigid arthoscope can be inserted through the Chung’s needle.
2. Ultra-thin rigid arthroscopy (Fig. 1)
  The ultra-thin rigid arthroscopy (Endostar-Nanoscopy System(Hanseo Medics Co.,Ltd., Seoul, Korea)) for TMJ procedure consists of 167 mm of total length, 57.5 mm of available length, a 0.8mm diameter fiber optic nanoscope, and a 16 Gauge Needle trocar with irrigation port and a Digital CCD Camera system with S/D card for recording, a 80W LED Light Source, and a 19-inch Kostec Color Video Monitor were used as the monitoring and recording system

### Surgical technique

The patient is prepared and draped in the standard fashion for arthrocentesis. The technique involves a conventional arthroscopic technique through the postero-lateral approach with Chung's needle (16 gauge needle size) under local anesthesia. Approximately 1.0-1.5 mL(pathologic TMJ about less than 1.0 ml) of lactated Ringer solution is injected into the superior joint space with Chung’s needle (16-gauge needle size). After pumping manipulation, closing the needle with 3 way coke for enlarging the superior joint space, and then another 16 G or 18 G needle inserts to the superior joint space for emitting the irrigation solution. With ultra-thin rigid arthroscopy view, joint space has been inspected and arthrocentesis was performed.

For the arthrocentesis, approximately more than 300 ml solution wash out the joint space and then ultra-thin rigid arthroscopy is inserted through the Chung’s needle for inspecting the joint space. When encountering the adhesion, resolve with trocar through the Chung’s needle and then instruct the mouth opening exercise during irrigation. When encountering the habitual dislocation, injure to the retrodiscal tissue by radiofrequncy (RF) surgery instrument.

After a sufficient range of movement has been achieved, the joint cavity is evaluated with arthroscopy. Finally, sodium hyaluronate (Seikagaku Kogyo Co, Tokyo, Japan) is injected into the superior joint space after joint irrigation, and removed the Chung’s needle. A mandibular motion exercise regimen was begun immediately after procedure.

## Case presentation

### Case 1

#### Closed lock (anterior disc displacement without reduction)

**Fig. 2:**
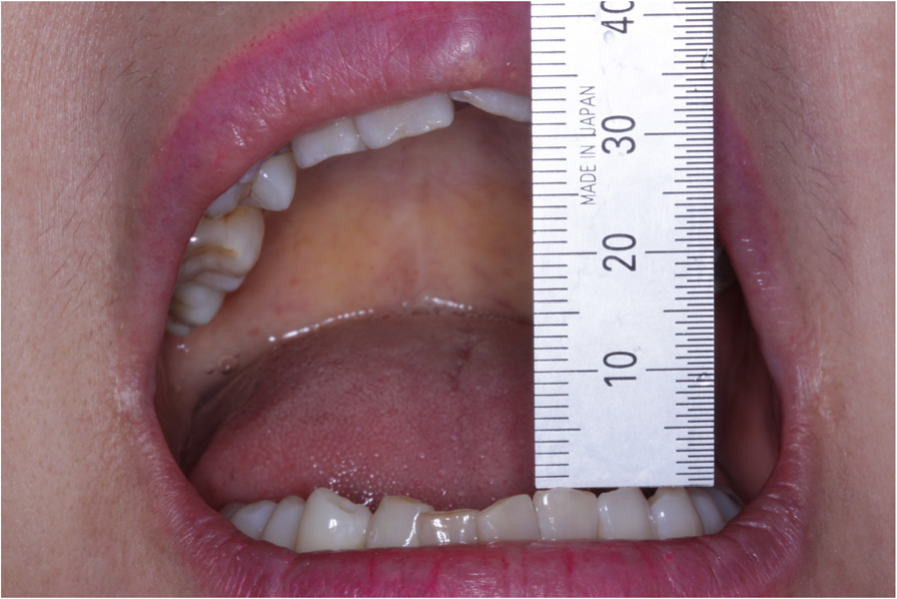
23 year-old female was complained of mouth opening limitation, TMJ pain and sound. The mouth opening at initial examination was 32 mm

**Fig. 3:**
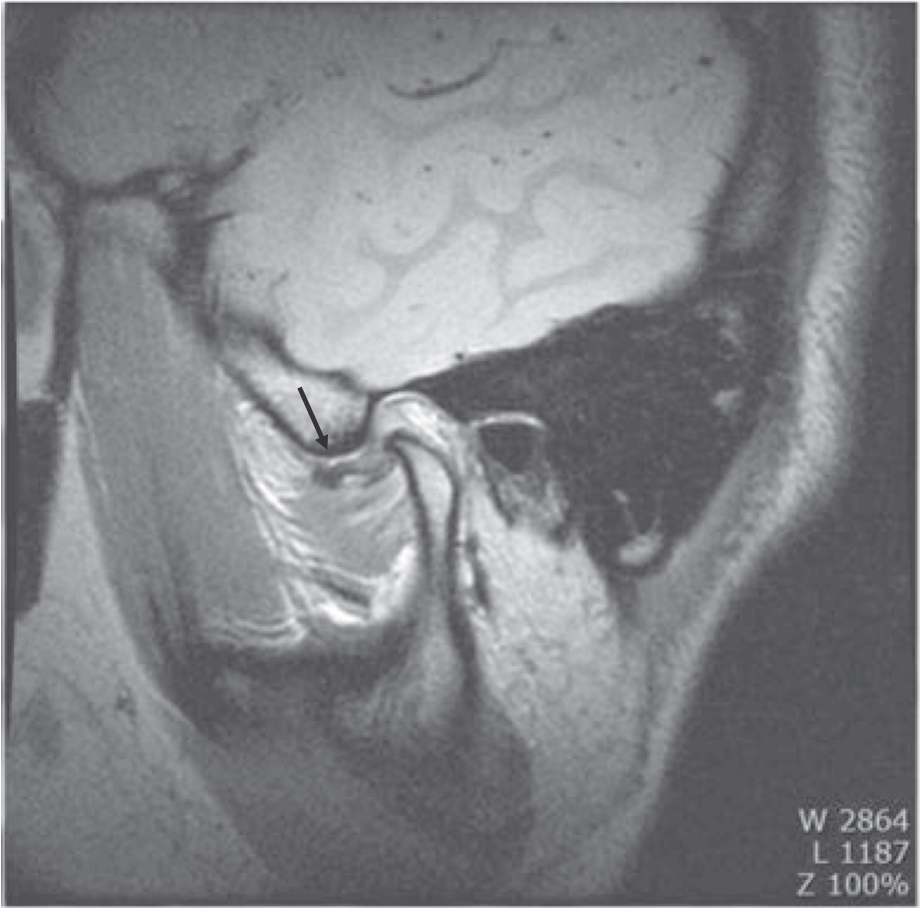
MRI shows the posterior band of the disc was anterior to the superior part of the condylar head. (Arrow indicates displaced disc anteriorly)

**Fig. 4:**
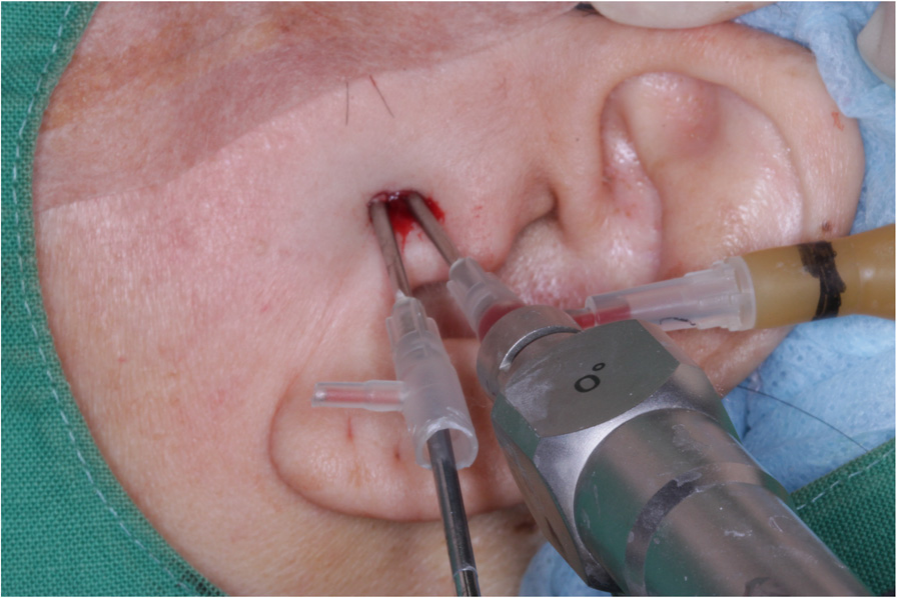
Ultra-thin arthroscopy inserted through the Chung’s needle and trocar was inserted through another portal

**Fig. 5:**
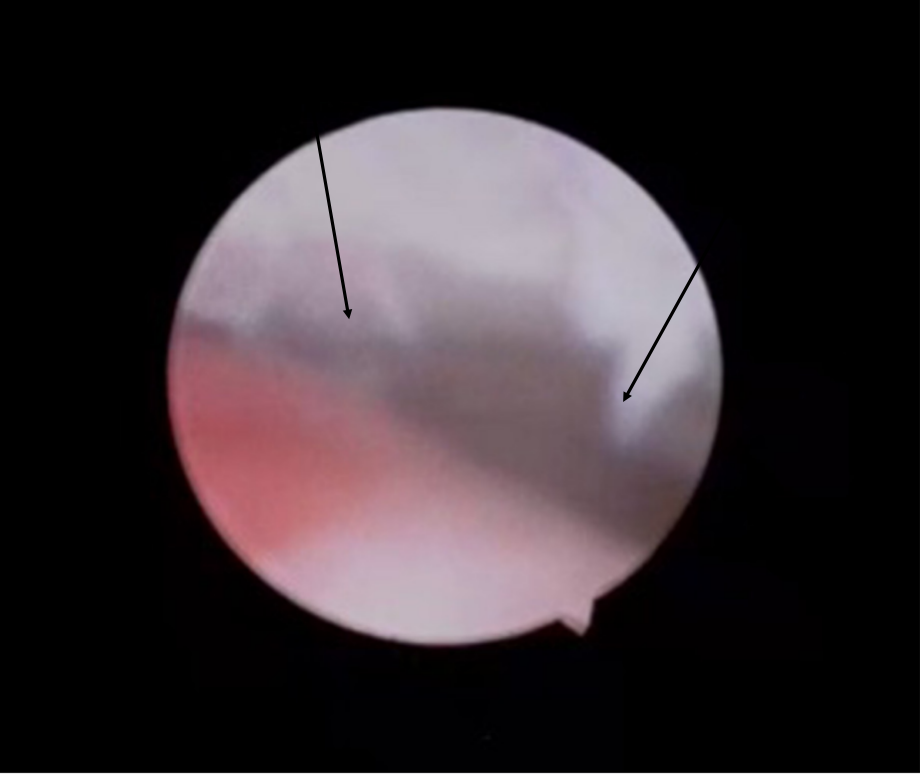
Adhesion was released with a blunt trocar under arthroscopic view. (Arrow indicates fibrous adhesion)

**Fig. 6:**
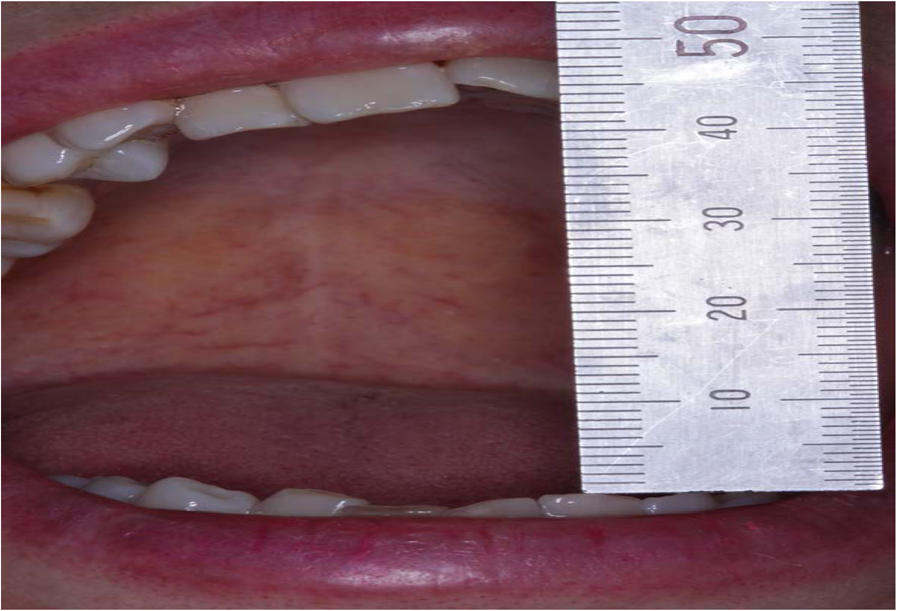
Range of mouth opening was improved from 32 mm to 45 mm on 7 days postoperatively

Twenty three year-old female was complained of mouth opening limitation, TMJ pain and sound. The mouth opening at initial examination was 32 mm (Fig. 2). The patient has suffered from this problem, and experienced arthrocentesis before. Under diagnosing to chronic closed lock on both TMJ and anterior disk displacement without reduction in MRI (Fig. 3), ultra-thin arthroscopic diagnosis and arthrocentesis was performed, and the adhesion was removed with trocar through the portal of Chung's needle (Figs. 4, 5). After arthrocentesis, range of mouth opening was 45 mm, and symptom was disappeared (Fig. 6).

### Case 2 (Habitual dislocation)

**Fig. 7:**
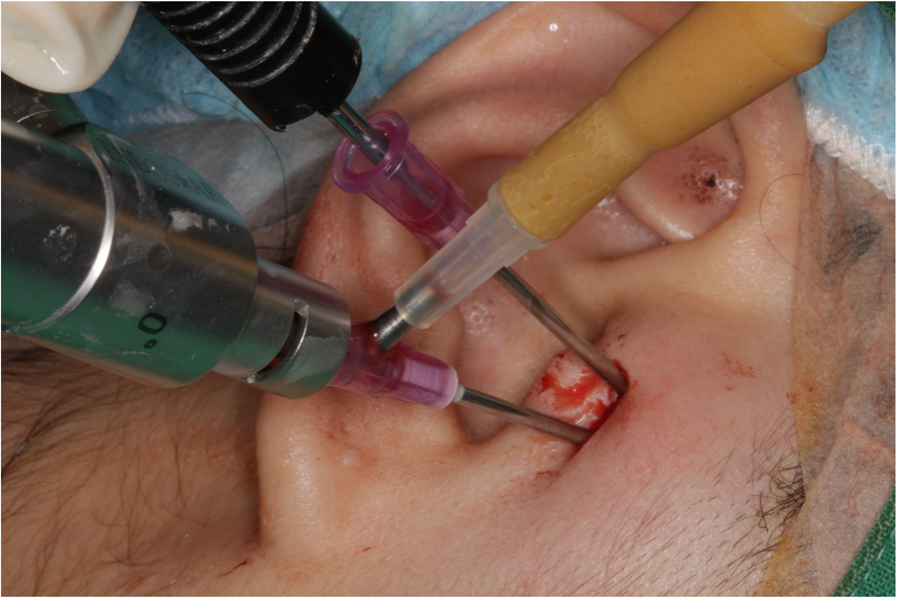
RF surgery tip inserted through a second portal

**Fig. 8:**
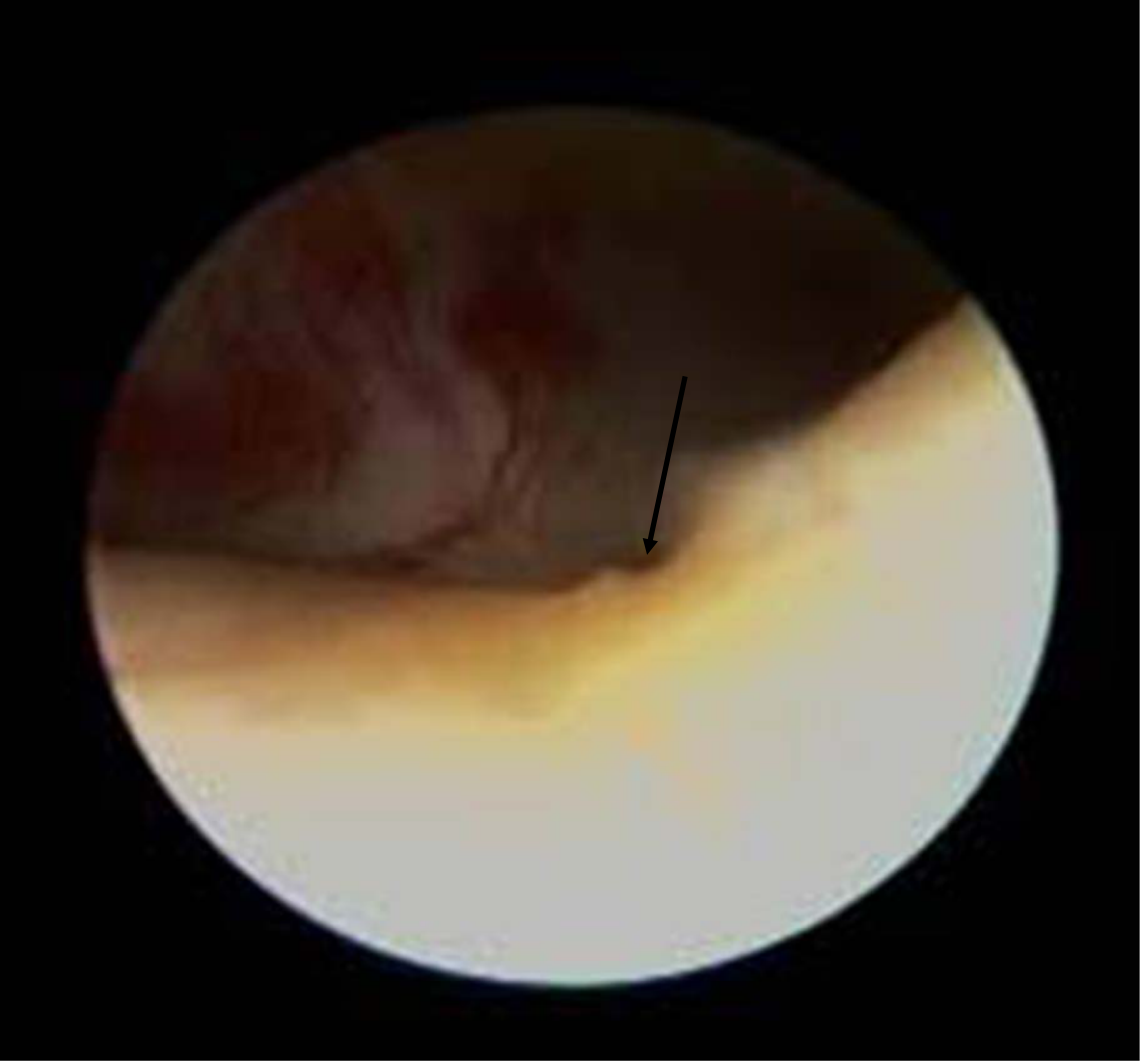
Retrodiscal tissue was injured with RF surgery instrument under arthroscopic view (Arrow indicates injured retrodiscal tissue)

Twenty two year-old female was complained of habitual dislocation, articular sound and pain. The patient had prior history of TMJ dislocation 3 to 4 times in a week, and reduced by herself. Chung’s needle introduced to the superior joint space of the TMJ, inserted the unltra-thin arthroscopy and RF surgery tip (Fig. 7) and retrodiscal tissue was injured with RF surgery instrument through the Chung’s needle and irrigated the joint space (Fig. 8). The patient had no pain during mouth opening and no episodes of dislocation after treatment.

## Discussion

This study suggests that the ultra-thin rigid arthroscopy showed highly detailed pathologic information of the intra-articular findings with high diagnostic accuracy. Arthrocentesis has been widely used, and several clinicians have reported on its efficacy and non-invasiveness [7].

The ultra-thin rigid arthroscopy with arthrocentesis has several potential advantages: it provides rapid and easy diagnosis and treatment simultaneously on outpatient basis. Iatrogenic damage is minimal and less bleeding, because of their small size. With this ultra-thin rigid arthroscopy puncture system, it is better to perform the arthrocentesis and diagnose the state of the TMJ than conventional arthroscopy.

Kalunian et al. [8] have described visually guided irrigation (VGIR) of the knee joint. They emphasized that visual guidance during joint irrigation was imperative not only for adequate irrigation of the different joint compartments, but also for the identification of morphologic characteristics that might be important in predicting the outcome. This visual guided irrigation system is similar to our concept in terms of performing arthrocentesis under arthroscopic view. This is a simple and comfortable procedure for the clinician and patient that would make use of a new ultra-thin arthroscopic system with Chung's needle. We thus were able to obtain reliable intra-articular images similar to conventional arthroscope images during combination with irrigation under local anesthesia. This minimally invasive technique also enabled us to compare between clinical outcome parameters and arthroscopic findings. Additionally, it can be used to inspect intra-articular status and treat pathologic conditions in the joint.

## Conclusions

In the present study, we examined morphologic changes in the superior joint space before and after procedure in patients with adhesion and habitual dislocation. Complications were few and patients would be satisfied. This ultra-thin arthroscopy could be used for the management of the TMJ disorders with minimal invasiveness.

## Consent

Written informed consent was obtained from the patient for publication of this Case report and any accompanying images. A copy of the written consent is available for review by the Editor-in-Chief of this journal.
